# Comparison of Oxytocin vs. Carbetocin Uterotonic Activity after Caesarean Delivery Assessed by Electrohysterography: A Randomised Trial

**DOI:** 10.3390/s22228994

**Published:** 2022-11-21

**Authors:** Ivana Paljk Likar, Emra Becic, Neza Pezdirc, Ksenija Gersak, Miha Lucovnik, Andreja Trojner Bregar

**Affiliations:** 1 Faculty of Medicine, University of Ljubljana, 1000 Ljubljana, Slovenia; 2 Division of Obstetrics and Gynecology, Department of Perinatology, University Medical Centre Ljubljana, 1000 Ljubljana, Slovenia

**Keywords:** electromyography, uterine contractions, caesarean section, haemorrhage, postpartum

## Abstract

Electrohysterography has been used for monitoring uterine contractility in pregnancy and labour. Effective uterine contractility is crucial for preventing postpartum haemorrhage. The objective of our study was to compare postpartum electrohysterograms in women receiving oxytocin vs. carbetocin for postpartum haemorrhage prevention after caesarean delivery. The trial is registered at ClinicalTrials.gov with the identifier NCT04201665. We included 64 healthy women with uncomplicated singleton pregnancies at term scheduled for caesarean section after one previous caesarean section. After surgery, a 15 min electrohysterogram was obtained after which women were randomised to receive either five IU of oxytocin intravenously or 100 μg of carbetocin intramuscularly. A 30 min electrohysterogram was performed two hours after drug application. Changes in power density spectrum peak frequency of electrohysterogram pseudo-bursts were analysed. A significant reduction in power density spectrum peak frequency in the first two hours was observed after carbetocin but not after oxytocin (median = 0.07 (interquartile range (IQR): 0.87 Hz) compared to median = −0.63 (IQR: 0.20) Hz; *p* = 0.004). Electrohysterography can be used for objective comparison of uterotonic effects. We found significantly higher power density spectrum peak frequency two hours after oxytocin compared to carbetocin.

## 1. Introduction

Postpartum haemorrhage (PPH) is the leading cause of maternal mortality and morbidity worldwide [1]. Up to 90% of cases of PPH are due to inadequate uterine contractility, known as uterine atony [1]. Uterotonics are used for PPH prevention as part of active management of the third stage of labour; the most widely used and recommended is oxytocin [2,3,4,5,6]. Several studies suggested that carbetocin, a synthetic analogue of oxytocin with a longer half-life, might be more effective for PPH prevention compared to oxytocin [2,7,8,9]. Studies of uterotonic efficacy are, however, limited by subjective estimation of blood loss or myometrial contractions after birth. At present, no tool in routine clinical practice allows accurate and objective assessment of postpartum myometrial activity and the efficacy of different uterotonic agents used for PPH prevention. Electrohysterography (electromyography of the uterus, EHG) is an objective, noninvasive method for the assessment of uterine contractility. It has been established by previous studies that the electric impulse of the myometrial smooth cells is responsible for myometrial contractions and can be detected in humans [10,11,12]. Myometrial electrical activity can be measured with electrodes directly placed on the uterus [13] or monitored noninvasively through the abdominal wall [14]. Measuring uterine electromyographic activity has similar effectiveness in the detection of uterine contractions to other methods, such as tocodynamometry or intrauterine pressure catheters [15,16].

Peripartal myometrium activity manifests as changes in several EHG parameters that can indicate different myometrial properties and uterine dynamics [17]. There are ‘time-related’ parameters such as electrical burst duration and interburst interval duration (inversely proportional to the frequency of bursts). These parameters represent the electrical equivalent of the duration and frequency of contractions. The second group is the ‘amplitude-related’ parameters, possibly representing EHG signal power or EHG signal energy. The third group of EHG parameters can be categorised as ‘frequency-related’. This includes the power density spectrum (PDS) median and peak frequency. PDS peak frequency has been one of the most predictive EHG parameters in both human and animal studies [18,19]. EHG signals are usually filtered in order to exclude most components of motion, respiration, and cardiac signals, which yields a narrow ‘uterine-specific’ band of 0.34–1.00 Hz. In the striated muscle EMG, the PDS covers a broad frequency range (20–400 Hz), with a more-or-less bell-shaped distribution of signal energy [20]. Other EHG parameters include EHG propagation velocity—calculated by the time interval between EHG signal arrivals at adjacent electrode pairs and the integral of the amplitudes of the PDS that corresponds to the PDS energy within the bursts of uterine electromyographic activity. Other non-linear electromyographic parameters have been also used for EHG signal characterisation, such as sample entropy, Lempel–Ziv, and time reversibility [21].

Several signal processing approaches have been developed and applied, including artificial intelligence-based tools dealing with EHG records or signals. For successful classification of EHG parameters, standard classifiers (*k* nearest neighbor, support vector machines, quadratic discriminant analysis) and advanced classifiers (variety of neural networks, random forests, and AdaBoost) are used. These new, complex signal processing techniques and classification methods allow for more information to be extracted from EHG signals and better numeric results. However, the use and implementation of these methods could lead to less clear physiological interpretation [22,23].

Traditionally, analysis of the EHG parameters was used for objectifying uterine dynamics [24,25], distinguishing true labour from false labour [17,26], or comparing preterm with term labour [27]. Only recently have studies regarding postpartum EHG been published [21,28]. However, no study to date used postpartum EHG to compare the efficacy of different uterotonic agents. The aim of our study was to compare the efficacy and objectively quantify the effect of carbetocin with EHG compared to the standard uterotonic oxytocin for postpartum haemorrhage prevention. For this purpose, we analysed different linear EHG parameters (PDS peak frequency, changes in PDS peak amplitude within EHG pseudo-bursts, the frequency and duration of EHG pseudo-bursts, and changes in the PDS integral) before and two hours after uterotonic administration. As we wanted to objectively assess blood loss, we also evaluated clinical laboratory parameters related to haemoglobin and haematocrit change before and after caesarean delivery.

## 2. Materials and Methods

### 2.1. Participants

This was a single-center, randomised, open-label trial conducted at the Department of Perinatology, Division of Gynaecology at the University Medical Centre Ljubljana, Slovenia from September 2020 to September 2021.

We included healthy women with uncomplicated singleton pregnancies at term (≥37 completed weeks of pregnancy) scheduled for elective caesarean section after one previous caesarean. All included women were not in labour. Women with contraindications for any of the study drugs (oxytocin or carbetocin), anaemia (haemoglobin level < 100 g/L), history of PPH, uterine fibroids, blood clotting disorder, placental disorder (placenta praevia or placenta accreta), hypertensive disorders of pregnancy, or renal, cardiac, or hepatic dysfunction were not included in the study.

### 2.2. Study Protocol

**Figure 1:**
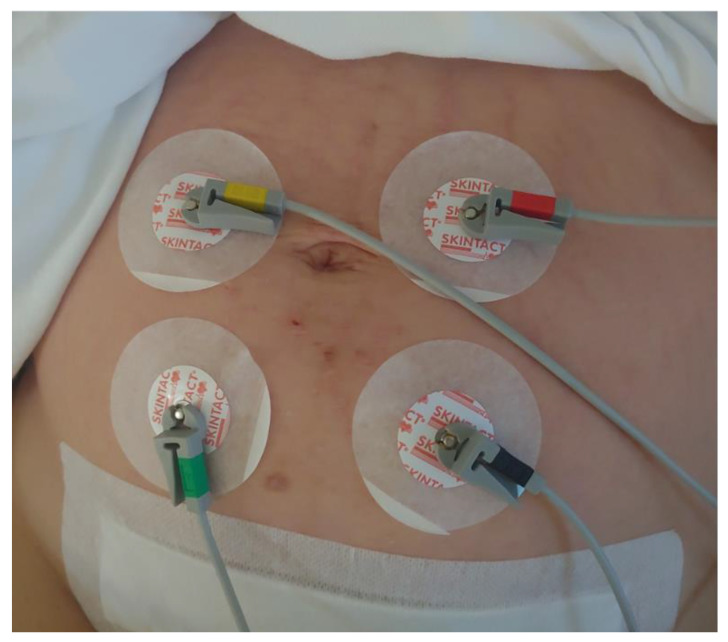
EHG electrode placement for recording after caesarean section—two sets of bipolar electrodes placed three to four centimeters apart around the navel.

**Figure 2:**
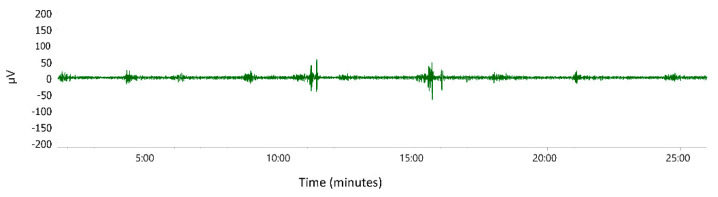
EHG recording with visible burst-like contractile elements (»pseudo-bursts«).

Directly following caesarean section, participants were admitted to a high dependency perinatal unit (HDU). A blood sample was obtained for a complete blood count and the first 15 min EHG was recorded before any uterotonics were administered. Women were then randomised in a 1:1 ratio in two groups. They received either 5 IU of oxytocin in a 100 mL saline solution as an intravenous bolus or 100 μg of carbetocin intramuscularly. Randomisation was conducted using sequentially numbered, opaque, computer-generated sealed envelopes. Treatments (oxytocin and carbetocin) were prepared and administered by HDU nurses independent of the study team. Study investigators and study participants were not blinded to the type of medication administered. After the randomly assigned drug was applied, a 30 min EHG recording was performed, followed by a third (30 min) EHG recording two hours after drug application. Electrohysterography recordings were performed with two sets of bipolar electrodes placed three to four centimeters around the navel, using a custom-built uterine EMG patient-monitoring system (SureCall Monitor, Reproductive Research Technologies, Houston, TX, USA), as shown in Figure 1. Women were asked to lay as still as possible in order to prevent any possible background noise while recording the EHG. Every movement or possible noise (coughing, breastfeeding, uterine massage, drug application) was carefully noted and excluded from further recording analysis. Labchart 8 software (ADInstruments, Castle Hill, Australia) was used for analysing the recordings. Signals were digitally filtered (band-pass 0.3–1 Hz) in order to exclude most components of motion, respiration, and cardiac signals from the analysis. Burst-like contractile elements (»pseudo-bursts«) in EHG results were identified by visual inspection of EHG signals, see the recording in Figure 2. We chose to use the same criteria to discern bursts of electrical activity and the same sampling frequency that we used in previously published work on EHG [14,17]. In order to include uterine-related electrical information, only groups of spikes with a mean voltage peak of >2 times the mean baseline voltage peak (separated in time by at least one minute) and occurring simultaneously with evidence of uterine contractions (as observed by women) were analysed. Data were sampled at 100 Hz. The power density spectrum (PDS) for 0.3–1 Hz frequencies of each pseudo-burst was obtained by using the fast Fourier transform. Cosine-bell windowing was applied in power spectrum calculations. The PDS curve is a function of frequency and represents the relative contribution of each frequency to the signal. We also analysed the integral of the amplitudes of the PDS corresponding to the PDS energy within the pseudo-burst. The PDS of all pseudo-bursts analysed in each recording were averaged to obtain a mean PDS peak frequency, PDS peak amplitude, and PDS integral for each woman. The frequency and duration of pseudo-bursts were also studied. Due to technical issues, propagation velocity of the EHG signal was not examined.

### 2.3. Parameters

Our primary outcome measure was the change in PDS peak frequency within EHG pseudo-bursts between recordings at admission (before uterotonic administration) and two hours after uterotonic administration. There were several secondary outcome measures investigated: changes in PDS peak amplitude within EHG pseudo-bursts, the frequency and duration of EHG pseudo-bursts, and changes in the PDS integral between EHG at admission and two hours after treatments. In our secondary outcome measures, we also included haematocrit (Ht) and haemoglobin (Hb) changes between admission to the HDU and 24 h after caesarean delivery. The process of weighing blood loss at the HDU was later discovered to be imprecise; therefore, it was omitted from the study.

### 2.4. Statistical Analyses

We used data from previous EHG studies on differences in PDS peak frequency in labour vs. non labour at term for sample size calculation. The reported difference was 0.56 − 0.44 = 0.12 Hz, with a standard deviation of 0.15 Hz [14]. A minimum sample size of 26 per group would be required to discern such difference in two postpartum uterotonic groups, calculated using a desired power of 0.80 and an alpha of 0.05. To ensure statistical soundness, we chose to include 32 women per group.

EHG parameters in the oxytocin and carbetocin group were compared using the Mann–Whitney U test. Background characteristics of the women were compared using the Student’s *t* test due to their normal distribution when used as numerical variables. Categorical variables were compared using the Pearson chi-square test or Fisher’s exact test. EHG parameters in the oxytocin and carbetocin groups were compared using the Mann–Whitney U test. For assessing the change in EHG parameters within each group at different times (before, right after, and two hours after uterotonic application), a Wilcoxon signed-rank test was used. For comparing the clinical laboratory parameters (Hb and Ht), we performed a Wilcoxon signed-rank test. Side effects were compared as categorical variables with the Pearson chi-square test. The program IBM SPSS Statistics for Windows, version 23.0 (IBM Corp., Armonk, NY, USA), was used for statistical comparison of groups. Statistical significance was determined at *p* < 0.050.

### 2.5. Ethics Statement

All women included in the study provided written informed consent for study participation. The National Medical Ethics Committee approved the study (project number 0120-398/2019/4, approved on 24 October 2019). The trial is registered at ClinicalTrials.gov with the identifier NCT04201665. The study protocol and details are available at: https://clinicaltrials.gov/ct2/show/NCT04201665 (accessed on 28 October 2022). The report follows the CONSORT recommendations for randomised control trials (equator-network.org, accessed on 28 October 2022).

## 3. Results

### 3.1. Participants

**Figure 3:**
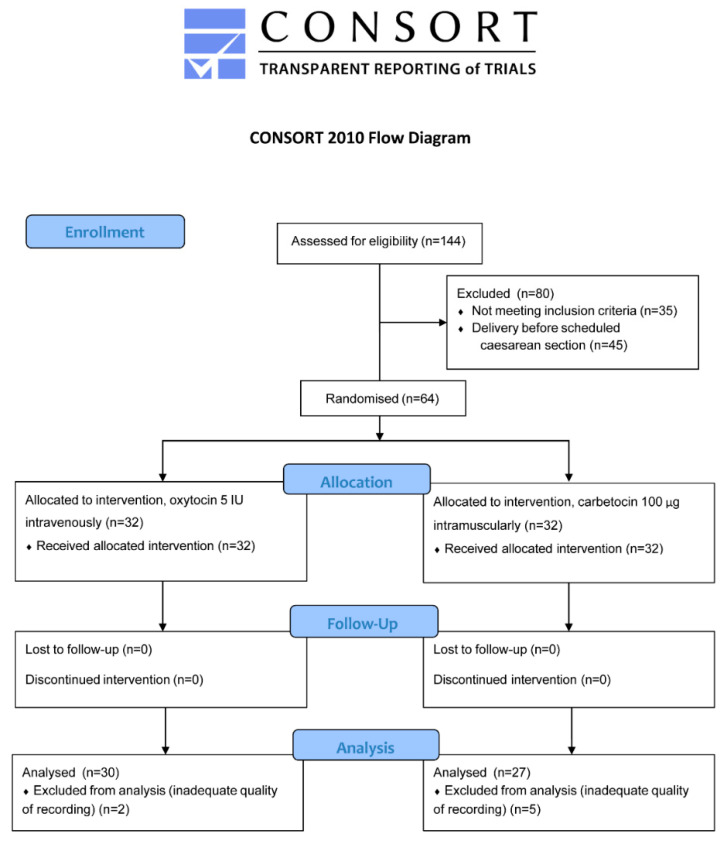
Randomisation and follow-up of study participants.

A total of 144 women were assessed for eligibility, and 64 were subsequently randomised (32 in the oxytocin and 32 in the carbetocin group) (Figure 3). After randomisation, two women in the oxytocin group and five women in the carbetocin group had an inadequate recording (too much background noise due to technical complications with electrodes while recording) and were subsequently excluded from analysis. A total of 30 women in the oxytocin group and 27 women in the carbetocin group remained for analysis. During EHG, six women (two from the oxytocin group and four from the carbetocin group) received additional uterotonics (ergometrine) due to clinically diagnosed increased blood loss or inadequate uterine contractility. The part of the EHG recording after ergometrine application in these cases was subsequently excluded from further analysis.

Women from both groups (oxytocin and carbetocin) had similar characteristics (Table 1). All included multiparous women had their previous delivery via caesarean section. There was one woman with a previous extrauterine pregnancy in the oxytocin group. No included women had a history of illicit drug abuse. Previous abdominal operations were also recorded. There was one woman with prior appendectomy (carbetocin group) and one with a prior inguinal hernial repair (oxytocin group). There were three women in the oxytocin group that had more than one previous gynaecological surgery (one woman had had a myomectomy, hysteroscopy, and laparoscopy, while two other women had had both a hysteroscopy and laparoscopy). There were two women who were subsequently discovered to have experienced myomectomy in the past and both received oxytocin.

### 3.2. Change in Power Density Spectrum (PDS) Peak Frequency

**Figure 4:**
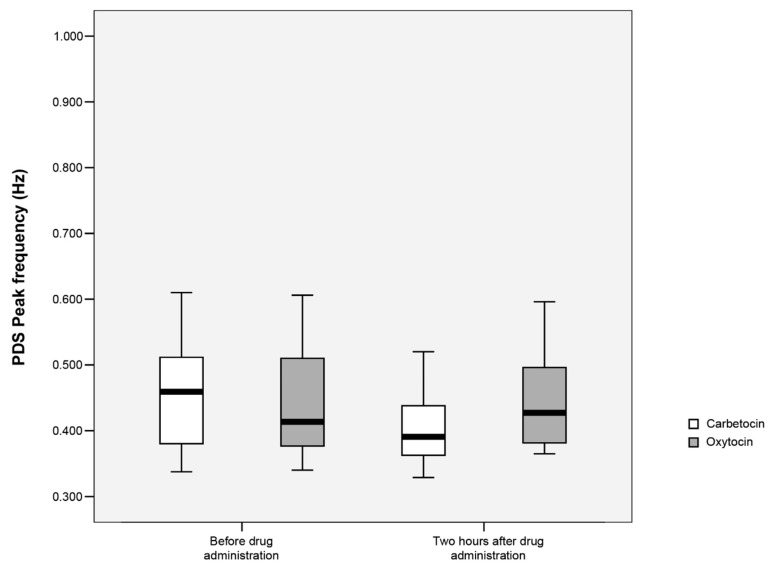
Power density spectrum (PDS) peak frequency of uterine electrohysterogram (EHG) pseudo-bursts in oxytocin and carbetocin groups before and two hours after drug application. Oxytocin PDS peak frequency before drug application: Mdn = 0.41 (IQR: 0.14) Hz; two hours after drug application: Mdn = 0.43 (IQR: 0.12) Hz. Carbetocin PDS peak frequency before drug application: Mdn = 0.46 (IQR: 0.13) Hz; two hours after drug application: Mdn = 0.39 (IQR: 0.08) Hz. Mdn: median. IQR: interquartile range.

We compared changes in PDS peak frequency from before drug application (recording time 15 min) to two hours after drug application (recording time 30 min) in the oxytocin vs. carbetocin group. Changes in PDS peak frequency were significantly smaller in the oxytocin compared to the carbetocin group (median (Mdn) = 0.07 (interquartile range (IQR): 0.87) Hz compared to Mdn = −0.63 (IQR: 0.20) Hz; *p* = 0.004). We observed significantly higher PDS peak frequency after two hours in the oxytocin (Mdn = 0.43 (IQR: 0.12) Hz) vs. carbetocin group (Mdn = 0.39 (IQR: 0.08) Hz), *p* = 0.030 (Figure 4).

PDS peak frequency was also compared within each group at three time points: before drug application, immediately after drug application, and two hours after drug application (recording time: 15 min, 30 min, and 30 min, respectively). In the carbetocin group, PDS peak frequency was lower (Mdn = 0.39 (IQR: 0.08) Hz) after two hours, compared to before drug application (Mdn = 0.46 (IQR: 0.13) Hz; *p* = 0.002). Similarly, PDS peak frequency after two hours was lower compared to PDS peak frequency immediately after drug application in this group (Mdn = 0.43 (IQR: 0.12) Hz; *p* = 0.045). There were no statistically significant differences in PDS peak frequency at the three time points in the oxytocin group. Table 2 presents a comparison of EHG parameters in the two study groups analysed as secondary outcomes. None of the EHG parameters differed significantly between groups at any time point.

### 3.3. Change in Haemoglobin and Haematocrit

We also compared haemoglobin (Hb) and haematocrit (Ht) values in the oxytocin vs. carbetocin groups using Wilcoxon signed-rank tests. Specifically, we compared the change in Hb and Ht values at admission to the HDU and 24 h after caesarean section. There was no statistically significant difference between the two groups regarding Hb change (Mdn = −4 (IQR: 0.10) g/L for oxytocin and Mdn = −6 (IQR: 9.5) g/L for carbetocin, *p* = 0.943) and no statistically significant difference for Ht change (Mdn = −14 (IQR: 31) % for oxytocin and Mdn = −11 (IQR: 47.25) % for carbetocin, *p* = 0.715). Comparison of admission Hb values in the oxytocin vs. carbetocin groups showed no statistically significant difference (Mdn = 117 (IQR: 14) g/L and Mdn = 113 (IQR: 9) g/L, respectively, *p* = 0.231). Similarly, the Hb values 24 h after caesarean section were not significantly different (Mdn = 112 (IQR: 12) g/L for oxytocin and Mdn = 113 (IQR: 1.5) g/L for carbetocin, *p* = 0.964).

Ht values at HDU admission also did not differ significantly between groups (Mdn = 34.9 (IQR: 13.9) % for oxytocin and Mdn = 33.6 (IQR: 3.9) % for carbetocin, respectively, *p* = 0.252). Similarly, there was no statistically significant difference in Ht values between groups at 24 h after caesarean section (Mdn = 33.3 (IQR: 4.5) % for oxytocin and Mdn = 32.8 (IQR: 4.1) % for carbetocin, respectively, *p* = 0.695).

### 3.4. Side Effects

The proportion of women experiencing any side effect after uterotonic administration was similar between the two groups (10 in the oxytocin vs. 8 in the carbetocin group; *p* = 0.757). A total of eight women (26.6%) receiving oxytocin had side effects defined as frequent oxytocin side effects, which included four women experiencing abdominal pain, one pruritus, two women experiencing tiredness, and one hypotension. In the carbetocin group, seven (25.9%) women experienced frequent side effects: six had abdominal pain, two had nausea and vomiting, and one pruritus. Shaking, which is defined as a rare side effect of the drugs used in this study, occurred in three women after oxytocin (10%) and in two women after carbetocin (7.4%) administration.

## 4. Discussion

The aim of our study was to compare the effect of oxytocin and carbetocin on postpartum uterine contractility after scheduled caesarean section with the use of EHG. We found no significant difference in EHG activity immediately following administration of the drugs used in this study. However, at two hours after administration, EHG PDS peak frequency was higher in the oxytocin group. A significant reduction in EHG peak frequency in the first two hours was observed after carbetocin but not after oxytocin administration.

Most recent Cochrane meta-analysis on the efficacy of different prophylactic uterotonic treatments postpartum showed an 8.7% absolute risk of PPH after vaginal birth (defined as ≥500 mL of blood loss regardless of its cause) with carbetocin, compared to 12.2% risk after oxytocin use [2]. Risk of PPH after caesarean section was also lower when carbetocin was used instead of oxytocin (44% vs. 60%) [2]. Moreover, for PPH ≥ 1000 mL, oxytocin plus misoprostol, oxytocin plus ergometrine, or carbetocin alone led to risk reductions of 12% to 17% compared with oxytocin [2]. These studies focused mainly on the clinical definition of PPH (estimated or measured blood loss, need for transfusion and additional uterotonics, Hb, Ht, and other laboratory parameters) and did not assess the effects of different uterotonics on uterine contractility per se. The need for additional uterotonics (assessed subjectively by the attending physician) was higher in the oxytocin group compared to the carbetocin group in our study. This is in line with previously published results [2,29]. However, when analysing only EHG data on uterine contractility, we found a significant reduction in uterine activity two hours after carbetocin administration, but not after oxytocin administration. These in vivo results are similar to in vitro findings by Cole et al. [30]. They found increased myometrial tissue contractility after oxytocin compared to carbetocin treatments [30]. It should also be mentioned that breastfeeding-related release of oxytocin might have influenced our results. It was previously discovered that synthetic oxytocin after prelabour caesarean section promotes endogenous release of oxytocin in response to skin-to-skin contact and neonatal suckling [31]. All women at our institution are encouraged to initiate breastfeeding as soon as possible after caesarean section. Therefore, we cannot exclude the possibility that the increased EHG PDS peak frequency found two hours after oxytocin administration was related to endogenous central release of oxytocin, which may have not developed in women who received carbetocin.

In the oxytocin group, two women (6.7%) were discovered to have had a myomectomy performed in the past. Myomas or uterine fibroids may alter myometrial contractility, leading to increased uterine irritability and contractility [32,33] and an increased risk of several obstetric complications [34,35]. We should note that the higher PDS peak frequency in the oxytocin group might be the consequence of preexisting surgically removed uterine myomas disrupting the endometrial contraction pathway. Previous EHG studies on scarred endometrium showed similar inter-channel correlation or propagation of the direction of EHG in labouring women with an intact scarred uterus and controls [36]. The two included cases had had subserosal and submucosal myomas removed, which should not have had a significant impact on the contracting myometrium.

EHG is a non-invasive monitoring methodology for uterine dynamics that has been extensively studied in pregnancy and during labour [17,37,38]. There are much less data on use of EHG as a means for assessing postpartum uterine activity. The feasibility of such EHG use has already been demonstrated by Diaz Martinez et al. [28]. They found uterine contractions after vaginal birth to be more frequent and more intense compared to caesarean section [21]. Both linear and non-linear postpartum EHG parameters have been studied, describing a lowering in signal amplitude and frequency after birth [21]. Our study focused exclusively on linear EHG parameters as the first study comparing the effects of two uterotonic treatments.

Our study has several limitations. The main outcome was not a clinical endpoint, i.e., blood loss or changes in Hb or Ht values after delivery, but was instead a marker of uterine contractility (EHG PDS peak frequency). The main goal of the study was, therefore, not to compare the clinical effects of different prophylactic uterotonics, which would require a much larger sample size. Instead, we focused on introducing a more objective assessment of the uterotonic effects of different treatments. Sample size was small and calculated on data from antepartum EHG recordings. The extrapolation might have led to insufficient sample size, which could explain the statistically non-significant differences in all secondary EHG parameters presented in Table 2. Small sample size could also be a source of type I statistical errors (i.e., finding a statistically significant difference in PDS peak frequencies when a true difference does not in fact exist). Moreover, this was a single-center trial. However, participant characteristics are similar to those reported in previous studies, allowing for the generalisation of our results.

Despite its several limitations, the study can be viewed as a first proof-of-concept study demonstrating the utility of postpartum EHG for assessment of the efficacy of different uterotonic treatments. For further studies, postpartum EHG analysis could be extended and also include non-linear EHG parameters (such as sample entropy, Lempel–Ziv, and time reversibility).

## 5. Conclusions

Our results show that the uterotonic effects of carbetocin after prelabour caesarean section diminish faster than those of oxytocin. A significant reduction in EHG PDS peak frequency in the first two hours was observed after carbetocin but not after oxytocin administration. The need for additional uterotonic use occurred more frequently in the oxytocin group. However, when comparing haemoglobin and haematocrit values and their change at admission to the HDU and 24 h after caesarean section, there was no significant statistical difference. Although the amount of analysed data is low, our results suggest the two uterotonics might be non-inferior in their effects. The compared side effects also showed no statistically significant difference and women from both groups were similarly affected. Our data, although limited, could lead to a better understanding of and improvement in dosage and frequency-of-administration protocols concerning uterotonic drugs in the future. A better understanding of in vivo uterotonic effects could lead to more cost-effective and tailored postpartum haemorrhage prevention and treatment. Future studies on postpartum EHG may focus mainly on non-linear EHG parameters. The application of artificial intelligence-based tools that are integrated with physiological responses could lead to the realisation of useful EHG-based clinical tools for postpartum haemorrhage prediction.

## Figures and Tables

**Table 1:** Background characteristics of the women (demographic data, reproductive data, perinatal data, surgery details (anaesthesia, surgeon, visual blood loss), pre-surgery laboratory values).

Characteristics	Oxytocin (*n* = 30)	Carbetocin (*n* = 27)	*p*
Age (years)	31.9 (4.5)	33.5 (5.1)	0.222 ^S^
Gestation (weeks)	39.7 (0.74)	39.8 (0.56)	0.643 ^S^
Parity			0.234 ^S^
1	19 (63.3%)	21 (77.8%)	
2 or more	11 (36.7%)	6 (22.2%)	
Pregnancy			0.792 ^P^
Second	19 (63.3%)	18 (66.7%)	
Third or more	11 (36.7%)	9 (33.3%)	
Previous miscarriage			0.764 ^P^
None	22 (66.7%)	19 (70.4%)	
1	6 (20%)	7 (25.9%)	
>1	2 (6.7%)	1 (3.7%)	
Body mass index (kg/m²)			
Before pregnancy	24.74 (4.9)	25 (4.92)	0.842 ^S^
At birth	29.8 (5.4)	29.9 (4.56)	0.937 ^S^
Weight (kg)			
Before pregnancy	67.8 (13.7)	69.5 (15.7)	0.668 ^S^
At birth	87.8 (14.6)	83.2 (14.2)	0.732 ^S^
Smoking	4 (13.3%)	0 (0%)	0.977 ^F^
Previous gynaecological surgery (other than caesarean section)	7 (23.4%)	5 (18.5%)	0.657 ^P^
Laparoscopy (endometriosis, adnexal surgery)	4 (13.3%)	1 (3.7%)	
Hysteroscopy (uterine septum removal)	6 (20%)	0	
Dilation and curettage (D&C)	2 (6.6%)	3 (11.1%)	
Myomectomy (subseros, submucosal myoma)	2 (6.6%)	0	
Large loop excision of the transformation zone (LLETZ)	2 (6.6%)	0	
None	23 (76.6%)	22 (81.2%)	
Haemoglobin level at preoperative counseling (g/L)	119.9 (9.6)	118.4 (9.3)	0.558 ^S^
Haematocrit level at preoperative counseling (%)	35.35 (2.64)	35.9 (2.68)	0.713 ^S^
Iron supplementation during current pregnancy	12 (40%)	9 (33%)	0.602 ^P^
Type of anaesthesia			0.258 ^F^
Local	24 (80%)	25 (92.6%)	
General	6 (20%)	2 (7.4%)	
Duration of anaesthesia (min)	55.6 (14.4)	63.7 (15.2)	0.045 *^,S^
Visual estimated blood loss during surgery (ml)			0.843 ^P^
300–400	24 (80%)	21 (77.8%)	
400–500	6 (20%)	6 (22.2%)	
Performing surgeon			0.843 ^P^
Specialist	13 (43.3%)	11 (40.7%)	
Resident in training	17 (56.7%)	16 (59.3%)	
Neonatal birth weight (g)	3518 (377)	3562 (356)	0.651 ^S^
Neonatal birth length (cm)	51.3 (2.2)	51.1 (1.6)	0.721 ^S^
Neonatal birth head circumference (cm)	35.7 (1.2)	35.1 (1.3)	0.048 ^S^
APGAR score			
1 min after birth	9	9	
5 min after birth	9	9	

Data are shown as mean and standard deviation or number and percentage. *p* value was calculated using a Student’s *t* test (^S^), Pearson chi-square (^P^) or Fisher’s exact test (^F^). * represents statistical significance (*p* ≤ 0.05).

**Table 2:** Secondary outcome measures and electrohysterography parameters.

EHG Parameters		Carbetocin		Oxytocin		
		Median	IQR	Median	IQR	*p*
At admission	PDS peak amplitude (Hz)	101.3	304.5	34.6	311	0.378
	Interval between pseudo-bursts (s)	400	428.6	420	302.5	0.642
	Pseudo-burst duration (s)	36.5	10.9	39.7	17	0.673
	Number of pseudo-bursts	3	3	2	1.8	0.202
	PDS integral (μV)	2.9	2.6	2.04	3.2	0.965
After drug application	PDS peak amplitude (Hz)	128.9	249.9	92.87	235	0.755
	Interval between pseudo-bursts (s)	415	375	450	540	0.061
	Pseudo-burst duration (s)	38.7	16.2	38.7	23.6	0.761
	Number of pseudo-bursts	5	6	4	3.3	0.304
	PDS integral (μV)	1.72	2.7	1.72	2.9	0.718
Two hours after drug application	PDS peak amplitude (Hz)	179.33	271.5	130.1	214	0.371
	Interval between pseudo-bursts (s)	360	326.5	300	386.3	0.570
	Pseudo-burst duration (s)	47.8	28.1	41.3	22.6	0.303
	Number of pseudo-bursts	5	4.3	4	5	0.961
	PDS integral (μV)	1.8	1.6	1.56	1.8	0.398

Data are shown as median and interquartile range (IQR). *p* value was calculated by statistical Mann–Whitney U tests.

## Data Availability

Not applicable.

## References

[1] Schlembach D., Helmer H., Henrich W., von Heymann C., Kainer F., Korte W., Kühnert M., Lier H., Maul H., Rath W. (2018). Peripartum Haemorrhage, Diagnosis and Therapy. Guideline of the DGGG, OEGGG and SGGG (S2k Level, AWMF Registry No. 015/063, March 2016). Geburtshilfe Frauenheilkd..

[2] Gallos I.D., Papadopoulou A., Man R., Athanasopoulos N., Tobias A., Price M.J., Williams M.J., Diaz V., Pasquale J., Chamillard M. (2018). Uterotonic Agents for Preventing Postpartum Haemorrhage: A Network Meta-Analysis. Cochrane Database Syst. Rev..

[3] Widmer M., Piaggio G., Nguyen T.M.H., Osoti A., Owa O.O., Misra S., Coomarasamy A., Abdel-Aleem H., Mallapur A.A., Qureshi Z. (2018). Heat-Stable Carbetocin versus Oxytocin to Prevent Hemorrhage after Vaginal Birth. N. Engl. J. Med..

[4] Moertl M.G., Friedrich S., Kraschl J., Wadsack C., Lang U., Schlembach D. (2011). Haemodynamic Effects of Carbetocin and Oxytocin given as Intravenous Bolus on Women Undergoing Caesarean Delivery: A Randomised Trial. BJOG Int. J. Obstet. Gynaecol..

[5] RG Guideline (2017). Prevention and Management of Postpartum Haemorrhage: Green-Top Guideline No. 52. BJOG Int. J. Obstet. Gynaecol..

[6] P Hemorrhage (2017). Committee on Practice Bulletins-Obstetrics Practice Bulletin No. 183: Postpartum Hemorrhage. Obstet. Gynecol..

[7] Lawrie T.A., Rogozińska E., Sobiesuo P., Vogel J.P., Ternent L., Oladapo O.T. (2019). A Systematic Review of the Cost-Effectiveness of Uterotonic Agents for the Prevention of Postpartum Hemorrhage. Int. J. Gynaecol. Obstet. Off. Organ Int. Fed. Gynaecol. Obstet..

[8] Taheripanah R., Shoman A., Karimzadeh M.A., Zamaniyan M., Malih N. (2018). Efficacy of Oxytocin versus Carbetocin in Prevention of Postpartum Hemorrhage after Cesarean Section under General Anesthesia: A Prospective Randomized Clinical Trial. J. Matern.-Fetal Neonatal Med..

[9] Elbohoty A.E.H., Mohammed W.E., Sweed M., Bahaa Eldin A.M., Nabhan A., Abd-El-Maeboud K.H.I. (2016). Randomized Controlled Trial Comparing Carbetocin, Misoprostol, and Oxytocin for the Prevention of Postpartum Hemorrhage Following an Elective Cesarean Delivery. Int. J. Gynaecol. Obstet..

[10] Vinken M.P.G.C., Rabotti C., Mischi M., Oei S.G. (2009). Accuracy of Frequency-Related Parameters of the Electrohysterogram for Predicting Preterm Delivery: A Review of the Literature. Obstet. Gynecol. Surv..

[11] Devedeux D., Marque C., Mansour S., Germain G., Duchêne J. (1993). Uterine Electromyography: A Critical Review. Am. J. Obstet. Gynecol..

[12] Garfield R.E., Maner W.L. (2007). Physiology and Electrical Activity of Uterine Contractions. Semin. Cell Dev. Biol..

[13] Wolfs G.M.J.A., van Leeuwen M. (1979). Electromyographic Observations on the Human Uterus during Labour. Acta Obstet. Gynecol. Scand..

[14] Trojner Bregar A., Lucovnik M., Verdenik I., Jager F., Gersak K., Garfield R.E. (2016). Uterine Electromyography during Active Phase Compared with Latent Phase of Labor at Term. Acta Obstet. Gynecol. Scand..

[15] Jacod B.C., Graatsma E.M., Van Hagen E., Visser G.H.A. (2010). A Validation of Electrohysterography for Uterine Activity Monitoring during Labour. J. Matern. Fetal Neonatal Med..

[16] Jezewski J., Horoba K., Matonia A., Wrobel J. (2005). Quantitative Analysis of Contraction Patterns in Electrical Activity Signal of Pregnant Uterus as an Alternative to Mechanical Approach. Physiol. Meas..

[17] Lucovnik M., Maner W.L., Chambliss L.R., Blumrick R., Balducci J., Novak-Antolic Z., Garfield R.E. (2011). Noninvasive Uterine Electromyography for Prediction of Preterm Delivery. Am. J. Obstet. Gynecol..

[18] Buhimschi C., Garfield R.E. (1996). Uterine Contractility as Assessed by Abdominal Surface Recording of Electromyographic Activity in Rats during Pregnancy. Am. J. Obstet. Gynecol..

[19] Maner W.L., Garfield R.E. (2007). Identification of Human Term and Preterm Labor Using Artificial Neural Networks on Uterine Electromyography Data. Ann. Biomed. Eng..

[20] Lucovnik M., Kuon R.J., Chambliss L.R., Maner W.L., Shi S.-Q., Shi L., Balducci J., Garfield R.E. (2011). Use of Uterine Electromyography to Diagnose Term and Preterm Labor: Uterine Electromyography for Diagnosing Labor. Acta Obstet. Gynecol. Scand..

[21] Mas-Cabo J., Ye-Lin Y., Garcia-Casado J., Díaz-Martinez A., Perales-Marin A., Monfort-Ortiz R., Roca-Prats A., López-Corral Á., Prats-Boluda G. (2020). Robust Characterization of the Uterine Myoelectrical Activity in Different Obstetric Scenarios. Entropy.

[22] Garcia-Casado J., Ye-Lin Y., Prats-Boluda G., Mas-Cabo J., Alberola-Rubio J., Perales A. (2018). Electrohysterography in the Diagnosis of Preterm Birth: A Review. Physiol. Meas..

[23] Jager F., Libenšek S., Geršak K. (2018). Characterization and Automatic Classification of Preterm and Term Uterine Records. PLoS ONE.

[24] Schlembach D., Maner W.L., Garfield R.E., Maul H. (2009). Monitoring the Progress of Pregnancy and Labor Using Electromyography. Eur. J. Obstet. Gynecol. Reprod. Biol..

[25] Rooijakkers M.J., Rabotti C., Oei S.G., Aarts R.M., Mischi M. (2014). Low-Complexity Intrauterine Pressure Estimation Using the Teager Energy Operator on Electrohysterographic Recordings. Physiol. Meas..

[26] Ye-Lin Y., Prats-Boluda G., Alberola-Rubio J., Bueno Barrachina J.-M., Perales A., Garcia-Casado J. (2013). Prediction of Labor Using Non-Invasive Laplacian EHG Recordings. Annu. Int. Conf. IEEE Eng. Med. Biol. Soc..

[27] Fergus P., Cheung P., Hussain A., Al-Jumeily D., Dobbins C., Iram S. (2013). Prediction of Preterm Deliveries from EHG Signals Using Machine Learning. PLoS ONE.

[28] Diaz-Martinez A., Mas-Cabo J., Prats-Boluda G., Garcia-Casado J., Cardona-Urrego K., Monfort-Ortiz R., Lopez-Corral A., De Arriba-Garcia M., Perales A., Ye-Lin Y. (2020). A Comparative Study of Vaginal Labor and Caesarean Section Postpartum Uterine Myoelectrical Activity. Sensors.

[29] Kang S., Zhou L., Zhu L., Wang Y., Yue Y., Yan L. (2022). Carbetocin versus Oxytocin for the Prevention of Postpartum Hemorrhage after Elective Caesarean Section in High Risk Women: A Prospective, Randomized, Open-Label, Controlled Trial in China. Clin. Exp. Obstet. Gynecol..

[30] Cole N.M., Carvalho J.C.A., Erik-Soussi M., Ramachandran N., Balki M. (2016). In Vitro Comparative Effect of Carbetocin and Oxytocin in Pregnant Human Myometrium with and without Oxytocin Pretreatment. Anesthesiology.

[31] Uvnäs Moberg K., Ekström-Bergström A., Buckley S., Massarotti C., Pajalic Z., Luegmair K., Kotlowska A., Lengler L., Olza I., Grylka-Baeschlin S. (2020). Maternal Plasma Levels of Oxytocin during Breastfeeding—A Systematic Review. PLoS ONE.

[32] Taylor E., Gomel V. (2008). The Uterus and Fertility. Fertil. Steril..

[33] Lee H.J., Norwitz E.R., Shaw J. (2010). Contemporary Management of Fibroids in Pregnancy. Rev. Obstet. Gynecol..

[34] Wallach E.E., Vu K.K. (1995). Myomata Uteri and Infertility. Obstet. Gynecol. Clin. N. Am..

[35] Szamatowicz J., Laudanski T., Bulkszas B., Akerlund M. (1997). Fibromyomas and Uterine Contractions. Acta Obstet. Gynecol. Scand..

[36] de Lau H., Yang K.T., Rabotti C., Vlemminx M., Bajlekov G., Mischi M., Oei S.G. (2017). Toward a New Modality for Detecting a Uterine Rupture: Electrohysterogram Propagation Analysis during Trial of Labor after Cesarean. J. Matern.-Fetal Neonatal Med. Off. J. Eur. Assoc. Perinat. Med. Fed. Asia Ocean. Perinat. Soc. Int. Soc. Perinat. Obstet..

[37] Euliano T.Y., Nguyen M.T., Darmanjian S., McGorray S.P., Euliano N., Onkala A., Gregg A.R. (2013). Monitoring Uterine Activity during Labor: A Comparison of 3 Methods. Am. J. Obstet. Gynecol..

[38] Vlemminx M.W.C., Rabotti C., van der Hout-van der Jagt M.B., Oei S.G. (2018). Clinical Use of Electrohysterography During Term Labor: A Systematic Review on Diagnostic Value, Advantages, and Limitations. Obstet. Gynecol. Surv..

